# Iron Mining for Erythropoiesis

**DOI:** 10.3390/ijms23105341

**Published:** 2022-05-10

**Authors:** Margherita Correnti, Elena Gammella, Gaetano Cairo, Stefania Recalcati

**Affiliations:** Department of Biomedical Sciences for Health, University of Milan, 20133 Milano, Italy; margherita.correti@unimi.it (M.C.); elena.gammella@unimi.it (E.G.)

**Keywords:** iron metabolism, erythropoiesis, erythroferrone, hepcidin, hypoxia

## Abstract

Iron is necessary for essential processes in every cell of the body, but the erythropoietic compartment is a privileged iron consumer. In fact, as a necessary component of hemoglobin and myoglobin, iron assures oxygen distribution; therefore, a considerable amount of iron is required daily for hemoglobin synthesis and erythroid cell proliferation. Therefore, a tight link exists between iron metabolism and erythropoiesis. The liver-derived hormone hepcidin, which controls iron homeostasis via its interaction with the iron exporter ferroportin, coordinates erythropoietic activity and iron homeostasis. When erythropoiesis is enhanced, iron availability to the erythron is mainly ensured by inhibiting hepcidin expression, thereby increasing ferroportin-mediated iron export from both duodenal absorptive cells and reticuloendothelial cells that process old and/or damaged red blood cells. Erythroferrone, a factor produced and secreted by erythroid precursors in response to erythropoietin, has been identified and characterized as a suppressor of hepcidin synthesis to allow iron mobilization and facilitate erythropoiesis.

## 1. Introduction

Iron is an element necessary for the survival of almost all living organisms, so the capacity of acquiring iron has been proposed as a significant driver of evolution on Earth [1]. In fact, it is required for oxygen transport and for the activity of enzymes involved in a variety of metabolic processes [2]. By cycling between the ferrous (Fe^2+^) and ferric (Fe^3+^) oxidation states, iron allows hundreds of proteins to function in many processes essential for life, including nucleic acid metabolism, energy production and neurotransmission. However, Fe^2+^ can promote Fenton-type reactions generating highly reactive oxygen species (ROS) that cause lipid peroxidation and DNA damage, eventually leading to cell death by ferroptosis [3,4,5]. Therefore, organisms develop precise regulatory mechanisms that tightly control iron homeostasis at both cellular and systemic levels to generate Fe^2+^ in limited concentrations and transiently.

## 2. Systemic Iron Homeostasis

Body iron balance is preserved through a system of proteins that coordinates duodenal iron absorption, recycling by reticuloendothelial (RE) macrophages, utilization (primarily by erythroid and proliferating cells) and storage (mainly in liver and RE cells). Despite these controlling mechanisms, human health is often affected because of too little or too much iron. Indeed, more than one billion people suffer from iron deficiency, which can often evolve into iron deficiency anemia. The latter is a huge global public health problem affecting more than 10% of the world’s population [6]. On the other hand, dysregulated iron uptake results in iron overload, such as in hereditary hemochromatosis, which is characterized by excess iron accumulation and consequent damage in many tissues (particularly liver, heart and endocrine glands) [7,8].

### 2.1. Iron Absorption

In the absence of a mechanism influencing iron excretion, which mainly results from unregulated mucosal lining and blood loss from the gastrointestinal tract, the control of intestinal absorption is a key determinant of body iron homeostasis [9,10]. Iron also cycles between the Fe^2+^ and Fe^3+^ states during its uptake, export and storage. Accordingly, absorption of the poorly available non-heme Fe^3+^ present in the diet requires the reduction by membrane-associated ferric reductase (Dcytb) and the subsequent transport of Fe^2+^ across the apical side of duodenal cells by divalent metal transporter 1 (DMT-1) [9].

Heme is an important source of iron because it is more bioavailable than non-heme iron, thereby largely compensating for its lower abundance in a standard diet. Several proteins have been proposed as transporters of heme across the intestinal brush-border membrane, but the precise mechanism of heme iron absorption remains not well understood [11], so heme trafficking without specific deliverers has been hypothesized [12].

Iron released from heme through the action of heme oxygenase 1 (HO-1) and non-heme iron is then exported from the enterocyte into the bloodstream by ferroportin (FPN) [13,14]. For the binding of iron to circulating transferrin, which delivers iron to all cells through the interaction with the transferrin receptor 1 (TfR1), its conversion to Fe^3+^ by two oxidases is necessary, the membrane-bound hephaestin and the circulating ceruloplasmin [9,10,15]. Under physiological conditions, intestinal iron absorption is controlled primarily by body iron content and erythropoiesis [16]. Specifically, iron uptake can be enhanced in the case of higher erythropoietic demand or suppressed when iron stores are repleted.

Duodenal iron absorption is regulated by both cell-autonomous and systemic signaling pathways. The local mechanism is centered on the interaction between iron regulatory proteins (IRPs), which post-transcriptionally regulate the expression of the major proteins of iron metabolism [17] and the iron- and hypoxia-sensing machinery based on hypoxia-inducible transcription factors (HIFs), in particular HIF-2α [18]. Indeed, under conditions of increased iron demand, HIF-2α can increase the expression of Dcytb, DMT-1 and FPN. At the systemic level, the IRP-1/HIF-2α interplay coordinates duodenal iron absorption according to systemic oxygen and iron availability. When iron levels in duodenal enterocytes are low, IRP1 activation leads to translational repression of HIF-2α, a mechanism aimed at maintaining transcriptional activation of iron transporters under control [18]. A similar dysregulation of the IRP-1/HIF-2α pathway, which links erythropoiesis and iron availability, leads to unabated expression of the HIF-2α-target gene erythropoietin (EPO) and polycythemia in rare patients with mutations in IRP1 [19] and IRP1-knockout mice [20].

However, the most important player in the communication of body iron requirements to the intestinal iron absorption sites is hepcidin, a short cysteine-rich peptide hormone generated and secreted mainly by hepatocytes [21]. Hepcidin exerts its regulatory function by binding to and downregulating the cellular iron exporter FPN. Upon binding, FPN is internalized, ubiquitinated, probably through the action of the recently identified E3 ubiquitin ligase RNF17 [22], and degraded [23]; the consequent block of iron flow, particularly from RE cells and duodenal enterocytes, decreases circulating iron levels [21]. Therefore, the hepcidin–FPN axis controls systemic iron homeostasis, and imbalances in this molecular circuit can lead to iron overload or deficiency conditions. Indeed, the removal or the overexpression of the hepcidin gene in genetically manipulated mice results in tissue iron overload and severe iron deficiency anemia, respectively. Importantly, altered hepcidin expression also leads to dysregulated iron homeostasis in humans [24]. When enhanced iron absorption is needed, hepcidin repression leads to FPN stabilization and increased iron export from the intestine to the bloodstream. Interestingly, the hepcidin-mediated and the local HIF-2α-dependent mechanisms collaborate, as iron efflux to the circulation results in iron deficiency in intestinal cells. Since low iron levels increase HIF-2α by decreasing prolyl hydroxylases activity, this results in a feed-forward cycle, favoring the expression of apical and basolateral iron transporters (reviewed by [25]).

The major use of iron, at least from a quantitative viewpoint, is in oxygen distribution. In fact, iron is an essential part of hemoglobin (Hb) (oxygen transport protein) and myoglobin (oxygen storage protein), and about 50% of body iron content is found in erythrocytes. As a component of Hb, iron represents a limiting factor in erythropoiesis, so, starting from erythroblast maturation, red blood cell (RBC) production increasingly depends on iron uptake. However, the role of a sufficient iron supply for the mitochondrial synthesis of iron-sulfur clusters and for the activity of several proteins that sustain the high proliferation rate of RBC precursors should not be underestimated. RE cell-mediated recycling of Hb-derived iron from the breakdown of senescent erythrocytes provides almost 80% of the considerable amount of iron (20–25 mg), which must reach the bone marrow daily [21]. Intestinal absorption of dietary iron accounts for the balance.

### 2.2. Regulation of Hepcidin Expression

Hepcidin expression is finely regulated at the transcriptional level by divergent stimuli, such as iron demand for erythropoiesis (the so-called erythroid regulator) and body iron stores (the so-called store regulator) or inflammation [10]. Given the aim of this review, we will briefly examine the mechanisms of hepcidin stimulation by iron and inflammatory conditions and discuss more in detail the pathways of hepcidin inhibition triggered by erythropoietic activity and hypoxia. For a detailed description of the control of hepcidin transcription, readers are referred to recent reviews [26,27].

#### 2.2.1. Hepcidin Regulation by Iron Availability

High iron availability stimulates hepcidin transcription through the bone morphogenic proteins (BMP)-SMAD1/5/8 transduction pathway. In response to enlarged liver iron stores, nonparenchymal cells, primarily sinusoidal endothelial cells, produce BMP2 and BMP6, which initiate heterodimerization between BMP type I (ALK2/ALK3) and type II receptors BMPRII/Act RIIA. This process results in the activation of the cytosolic SMAD1/5/8 complex and SMAD4, eventually increasing hepcidin expression [27]. Hemojuvelin (HJV) functions as a BMP coreceptor and is critical for hepcidin expression in response to iron loading [28], as shown by the severe iron overload (juvenile hemochromatosis) developed by patients with mutations in HJV. In addition, BMP-dependent activation of hepcidin transcription is modulated by a complex comprising human hemochromatosis protein (HFE) and transferrin receptor 2 (TfR2). The lack of these proteins in mouse models leads to iron overload, and mutations in the corresponding genes are present in patients with distinct types of hemochromatosis (reviewed by [28,29,30,31]). Other liver proteins such as furin [32] and neogenin [33] interact with HJV to favor the proper assembly of BMPs/BMPR complexes through still incompletely understood pathways. Conversely, when iron levels fall, these pathways are inhibited mainly thanks to the action of matriptase-2 (TMPRSS6), which cleaves HJV from the plasma membrane, thereby preventing its coreceptor function. Further hepcidin inhibition is due to competition for BMPs by the cleaved soluble fragment (s-HJV), which acts as a decoy molecule [34,35,36]. The key role of TMPRSS6 was demonstrated by the strong hepcidin increase, followed by impaired iron absorption and anemia, caused by its inactivation in mice [28,29,30,31]. Notably, mutations in the TMPRSS6 gene underlie a rare form of hypochromic microcytic anemia unresponsive to oral iron therapy [37]. Moreover, since heparin binds BMP and inhibits hepcidin synthesis, endogenous heparan sulfates play a role in the control of hepcidin expression [38].

#### 2.2.2. Hepcidin Regulation by Inflammatory Stimuli

Since a considerable amount of iron is required daily for Hb synthesis and erythroid cell proliferation, the erythropoietic compartment is a privileged iron user. However, as fundamental though efficient oxygen transport is, the need of limiting microbial growth represents another relevant constraint in evolution, as pathogens desperately require iron. This goal is achieved also by increasing hepcidin levels in response to inflammation and infection, a defense mechanism that protects the host from pathogens by restricting iron availability [39]. On the other hand, in chronic inflammation, persistently elevated cytokine levels result in prolonged hepcidin activation and decreased iron bioavailability, which is a major contributor to the so-called anemia of inflammation (AI). At the molecular level, hepcidin is induced by inflammatory mediators, in particular interleukin 6 (IL-6) [40], which triggers hepcidin transcription mainly via the JAK–STAT3 signaling pathway, though inflammation may stimulate hepcidin also through TGF-β/BMP superfamily ligands, such as Activin B [41]. Since a lack of iron can lead to AI, but iron supplementation may exacerbate the risk of infections, understanding the central role of hepcidin in the interplay between iron handling, anemia and infections is of paramount importance.

## 3. Crosstalk between Iron and Erythropoiesis

The HIF system is the most important mediator of cellular adaptation to insufficient oxygen. HIF1α and HIF2α induce the transcription of a large number of genes involved in the response to hypoxia both at the cellular and organismal levels. HIF2α-mediated induction of EPO [42,43] is the systemic response to hypoxia, which eventually re-establishes optimal oxygen supply to tissues [44]. EPO binding to its receptor promotes survival, proliferation and differentiation of erythroid progenitors through the Jak2-Stat5 pathway, ultimately leading to an increased number of circulating RBCs. Given the role of iron in erythropoiesis, genes coding for proteins directly or indirectly involved in iron metabolism are included in the variety of HIF target genes; in particular, as reported above, HIF2α upregulates the expression of proteins required for iron trafficking [18]. Several recent studies highlighted a direct EPO-dependent mechanistic connection between erythropoiesis and iron homeostasis aimed at assuring the needed amount of iron to sustain the enhanced RBC production. Indeed, differentiating erythroid precursors show a very high surface display of TfR1 (about 800,000/cell) to internalize transferrin-bound iron. Remarkably, mice with deletion of Stat5, which suffer from microcytic anemia, showed a strong decrease in TfR1-mediated iron uptake and Hb synthesis, as the Jak2-Stat5 pathway also controls the transcription of TfR1 and IRP2 [45].

Under conditions of low iron availability, the regulatory mechanisms that maintain the balance among the various components of Hb on the one hand suppress EPO production via the IRP1-HIF2α axis to prevent disproportionate iron use by erythropoiesis and on the other hand also curb erythroid maturation by inhibiting aconitase [20]. Moreover, through a pathway involving the iron-sensing TfR2, erythroid iron restriction represses Scribble, a regulator of receptor trafficking, thereby altering the response to EPO [46]. Using mice with specific deletion of TfR2 in erythropoietic cells, it has also been shown that TfR2 modulates the sensitivity of erythroblasts to EPO [47], thereby indicating a role for TfR2 in the crosstalk between iron homeostasis and RBC production. The link between iron and erythropoiesis was also substantiated by a study showing that the inhibition of hepcidin, which underlies testosterone’s stimulation of erythropoiesis is iron-dependent [48].

### 3.1. Hepcidin Regulation by Erythropoiesis

**Figure 1:**
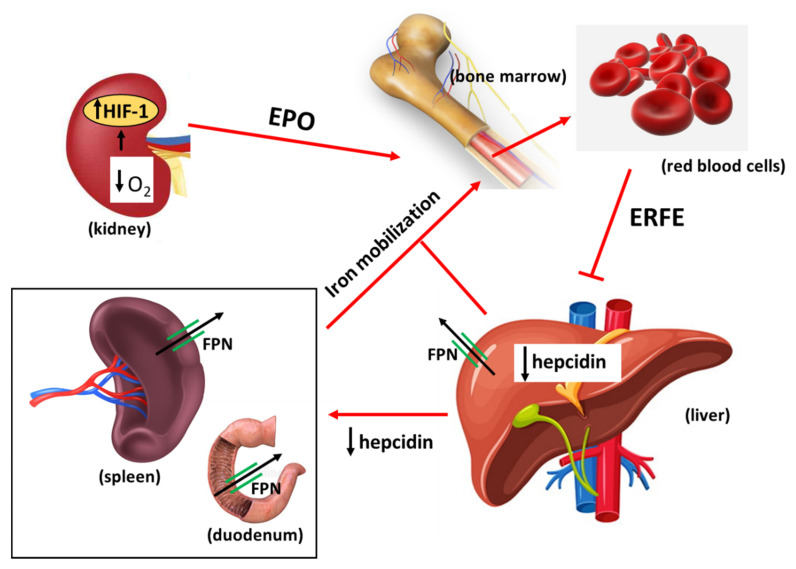
Major regulatory pathways involved in the effect of erythropoietic stimulation on the regulation of systemic iron homeostasis.

Studies in animal models showed that at the systemic level erythropoietic activity and iron homeostasis are coordinated by hepcidin; when RBC production is enhanced, hepcidin downregulation increases both duodenal iron uptake and release of iron from RE macrophages and liver stores (Figure 1) [49]. Importantly, these findings were confirmed in thalassemic patients, in which higher EPO concentrations and biomarkers of sustained erythroid activity were associated with hepcidin suppression [50]. It should be noted that hepcidin inhibition triggered by the “erythroid regulator” appears to prevail over other pathways that induce hepcidin. Indeed, inflammation-dependent signaling in mice was overcome by erythropoietic drive [51], and dysregulated erythropoiesis was able to inhibit hepcidin even under conditions of iron overload [52].

Decreased oxygen availability caused by hypoxia or compromised oxygen transport/delivery induces HIF-1 dependent erythropoietin (EPO) synthesis in the kidney. Increased circulating EPO levels stimulate erythroid activity in the bone marrow, which is accompanied by higher expression of erythroferrone (ERFE) by erythroid precursors. By interfering with the BMP signaling pathway, ERFE downregulates hepcidin production in the liver. In turn, the lower hepcidin levels permit ferroportin (FPN)-dependent iron release into plasma out of the iron recycling (spleen macrophages) and iron absorption (duodenal enterocytes) compartments. The higher iron availability in the bloodstream satisfies the enhanced needs for red blood cell production.

### 3.2. The Role of Hypoxia

The role of hepcidin as a key regulator appears well established, but where does the iron needed to sustain a massive increase in erythropoietic drive come from?

Optimization of iron transport mechanisms, including increased intestinal absorption and fast and efficient recycling of iron derived by processed RBCs, can provide iron to the erythroid bone marrow. Indeed, it has been shown that in healthy volunteers exposed to hypoxemic conditions (rapid ascent to 4559 m) hepcidin is repressed, and duodenal iron transport in biopsy specimens is rapidly upregulated [53]. These changes aimed at ensuring a sufficient iron supply for hypoxia-induced compensatory erythropoiesis may be accompanied by a reduction in iron stores. In fact, in mice overexpressing human EPO, excessive erythrocytosis was associated with reduced transferrin saturation and low iron stores [54]. Similarly, studies in which human subjects were acutely exposed to low oxygen as a consequence of rapid [53,55] or relatively slow [56,57] ascent to very high altitudes showed that increased erythropoiesis was accompanied by a massive mobilization of iron from stores. These data are in line with the iron deficiency found in polycythemia vera patients presenting with excessive erythrocytosis [58]. Therefore, the mechanisms underlying the short-term adaptation of iron metabolism to high-altitude hypoxia are characterized by a hepcidin-regulated increase in iron absorption and decrease in iron storage, which provides iron to meet the higher requirements [53,55,56,57].

It is less well established whether and how iron metabolism is regulated in residents at high altitude who are chronically exposed to low oxygen [59]. More than 20 million people worldwide live at high altitude (above 3000 m) and face several clinical problems [60]; most of these individuals are residents of the Andean region of South America. The oxygen homeostasis pathways respond to the stress of acute or chronic high-altitude hypoxia in ways that facilitate peripheral oxygen delivery. The various highlands populations acclimatized successfully by means of different physiological mechanisms, and Andean people adapted mainly by increasing Hb production [61]. Indeed, it has been recently demonstrated that excessive erythrocytosis in Peruvian high-altitude residents is associated with impressive total red blood cell volume expansion and very high Hbmass [62]. However, the normal ferritin levels and transferrin saturation found in Puno residents (3400 m) [63] indicated a lack of significant losses of stored iron. These results are also consistent with the greater prevalence of iron deficiency in lowlanders than in adapted Tibetan Sherpa upon ascent to 5050 m [57]. Similarly, Ethiopian highland subjects of Amhara and Oromo ethnicities did not show decreased body iron stores despite significantly higher Hb concentration [64]. Since hepcidin was not suppressed under steady-state hypoxia, the authors hypothesized that the response was possibly due to the stable erythropoietic drive caused by chronic exposure to hypoxia, in contrast with acute elevation of erythropoietic iron requirements [64]. Though the mechanisms providing more iron in these subjects chronically exposed to hypoxia remain unexplained, the available data indicate an evolutionary adaptation against iron deficiency. Despite adaptive mechanisms, a significant proportion of high-altitude residents develop a clinical syndrome known as chronic mountain sickness (CMS), which is mainly characterized by chronic and severe hypoxemia, excessive erythrocytosis and pulmonary artery hypertension (PAP) [65]. Since PAP is inversely related to iron stores/availability [66], it is tempting to speculate that sparing iron stores represents a defense against CMS for long-time residents at very high altitude, though additional studies are necessary to validate this hypothesis.

Regarding the molecular mechanisms underlying hepcidin repression under conditions of enhanced erythropoietic activity, though a study proposed HIF1α-dependent repression of hepcidin transcription [67], subsequent findings [68,69] did not confirm a direct suppressing effect of hypoxia/HIF1 on hepcidin transcription and showed that the role of HIF in this context is to stimulate EPO production. However, the expression of proteins that negatively control hepcidin expression, such as Matriptase2 [70], furin [32,71] and platelet-derived growth factor BB [72], is induced by HIF-1/2α. The possible complementary role of these pathways in hepcidin downregulation in these settings remains to be completely understood.

Initial studies showing (1) strong downregulation of hepcidin in mice injected with EPO [18,52,73], (2) inhibition of hepcidin transcription in EPO-treated cell lines [74,75] and (3) a prompt decrease in hepcidin levels [76,77] that preceded any significant change in potential mediators following EPO administration to healthy human subjects [77,78] have suggested that EPO could directly repress hepcidin expression. However, other evidence has been obtained against a direct effect of EPO in hepcidin inhibition. In fact, hepcidin levels are not decreased in mice with damaged bone marrow (BM) [79,80] and in patients with Diamond–Blackfan anemia, which is characterized by erythroid hypoplasia [81]. Therefore, these results strongly indicate that hepcidin downregulation is not directly controlled by EPO and instead requires the intervention of an active erythroid marrow. Moreover, a report showing that the repressing effect of EPO on hepcidin was present also in mice lacking EPO receptor in liver cells [82] rules out that EPO binding to liver receptors is involved in hepcidin suppression.

Having excluded a direct influence of EPO, the search for effectors connecting erythropoiesis with liver hepcidin downregulation has been pursued. Among the candidates proposed as potential “erythroid factors”, the role of growth differentiation factor 15 (GDF15), a member of the transforming growth factor-β superfamily, and twisted gastrulation factor 1 (TWSG1) have been suggested but not confirmed, and the hepcidin suppressing capacity of PDGF-BB [72] should be investigated further (discussed in [83]).

## 4. Erythroferrone

The hormone erythroferrone (ERFE) appears to be the major suppressor of hepcidin expression in response to higher erythropoietic activity and EPO, i.e., the erythroid regulator hypothesized by Finch (Figure 1) [16]. It has been shown that ERFE is mainly expressed by erythroblasts in response to EPO treatment, and its induction depends on the JAK2-STAT5 pathway [52]. However, ERFE is also expressed in skeletal muscle and adipose tissue and is a member of the C1Q/tumor necrosis factor (TNF)-related protein family (CTRP15). Indeed, the Fam132b mRNA coding for ERFE has been previously identified as a transcript coding for a protein originally called myonectin [84]. Recently, an interesting study demonstrated considerable EPO-independent production of ERFE by osteoblasts. By inhibiting bone resorption, ERFE protects the bone, and thus, this mechanism may represent a complementary way to favor expanded erythropoiesis [85]. Regarding the molecular basis underlying the action of ERFE on hepcidin repression, it has been shown that ERFE downregulates hepcidin transcription by binding to BMPs, thereby inhibiting the interaction with their hepatic receptor and the downstream BMP/SMAD signaling [52,86].

The role of ERFE in basal erythropoiesis was indicated by studies in ERFE knockout mice, which did not show abnormalities in iron and hematological parameters at baseline. However, they were not able to promptly suppress hepcidin in response to erythropoietic stress [73]. On the other hand, we showed that in healthy humans, ERFE responds even to very low EPO doses not associated with Hbmass expansion, a functional marker of erythropoietic response [87]. As such, ERFE appears a suitable marker to be included in the Athlete Biologic Passport for the detection of EPO abuse and could represent an advance in the antidoping field. Moreover, we recently showed in humans that ERFE levels are enhanced by exposure to high altitude (3800 m) for 15 h, thereby indicating the role of ERFE in acute adaptation to hypoxia [87]. Increased ERFE levels were also found after a longer period of time spent at a more moderate altitude [88]. Overall, it seems that ERFE function is not restricted to stress erythropoiesis, as previously supposed on the basis of the first studies [89].

ERFE plays a relevant role also in pathological settings involving altered erythropoiesis; in particular ERFE induction was found in various types of mouse anemia [90], and ERFE plasma levels are elevated in patients with hereditary hemolytic anemias [91]. Remarkably, high induction of ERFE expression has been observed in anemias with ineffective erythropoiesis, such as β-thalassemia, which are characterized by an expanded erythroblast compartment [92]. Interestingly, deletion of ERFE improved iron overload and ineffective erythropoiesis in a mouse model of thalassemia [93]. Even more severe anemia and iron overload can be caused by ERFE variants either due to aminoacid substitution in ERFE, such as in patients with congenital dyserythropoietic anemia type II [94] or in splicing factors generating alternative ERFE transcripts, such as in subjects affected by myelodysplastic syndromes, a group of disorders generally presenting with anemia and iron overload [95]. In both cases, very high plasma concentrations of a variant ERFE that maintained the capacity to suppress hepcidin were found.

ERFE may also be important in AI. Studies in mice lacking ERFE showed that AI caused by injection of inactivated bacteria was more severe than in control animals and was associated with higher hepcidin levels and iron sequestration, thus suggesting that ERFE antagonizes AI [96]. However, ERFE is not able to override the inflammatory induction of hepcidin. A similar situation with elevated levels of both hepcidin and ERFE has been recently described in patients with AI caused by Mycobacterium tuberculosis infection [97]. Notably, following treatment, hepcidin decreased rapidly along with inflammatory markers, whereas ERFE showed a slower decline. The persistence of its inhibitory effect could thus possibly increase iron availability and contribute to the resolution of anemia. Using a mouse model with different levels of ERFE overexpression, it has been recently shown that ERFE excess leads not only to iron overload but also impacts BMP-mediated development [98]. These findings provide insights into the molecular mechanisms leading to the developmental abnormalities that sometimes accompany the severe forms of iron loading anemias with ineffective erythropoiesis.

The absence of an apparent phenotype in ERFE knockout mice under physiological conditions suggests the possible existence of other unidentified regulators linking the erythroid compartment with hepcidin expression in the liver. For example, it has been demonstrated that erythroid TfR1 is an ERFE-independent inhibitor of hepcidin in response to expanded erythropoiesis [99].

## 5. Conclusions and Future Directions

In recent years, several key aspects of the regulatory system by which the erythroid compartment, which consumes the largest share of circulating iron, controls iron trafficking to obtain iron for Hb synthesis have been unraveled. ERFE produced by erythroid precursor cells is currently the best-characterized hepcidin inhibitor, and mechanistic insights into its interaction with the hepatic BMP/SMAD pathway have been provided. Given the role of dysregulated ERFE production in hematologic disorders, manipulation of ERFE levels may represent an innovative therapeutic approach for diseases such as ineffective erythropoiesis with iron overload or AI. The characterization of the mechanism based on the ERFE-hepcidin–FPN axis by which systemic iron homeostasis is modified has seen considerable advances. However, other mechanisms based on local iron supply for the development of erythroid precursors in the BM may play a relevant role in this context.

In the BM, erythroblastic island macrophages (EIMs) are surrounded by erythroid progenitors, adhere to erythroblasts, favor proliferation and differentiation and phagocytose extruded nuclei, thus providing an environmental niche for RBC production [100]. Since EIMs are able to degrade heme and recycle iron [101], the possibility that EIMs could directly provide iron to erythroblasts has been envisaged. Indeed, EIMs express high levels of FPN [101], which is induced by SpiC [102], a transcription factor also essential for EIM differentiation, EI formation and RBC production [103]. Moreover, the existence of iron-recycling machinery in EIMs has been recently documented [104]. Such a mechanism, in which EIMs function as iron-rich nurse cells and use FPN to export iron, thus providing a prompt and direct supply of iron, may be particularly relevant when erythropoiesis is overactive and TfR1-mediated internalization of transferrin, which has become iron saturated in the gut and in the spleen, may not be able to provide enough iron to erythroblasts.

The situation might be even more complex; in fact, a recent study has shown that iron availability in the BM is relevant for the development of other hematopoietic precursors, as it controls hematopoietic stem cells (HSCs) self-renewal vs. differentiation decisions [105]. Under stress conditions, microbiota-produced butyrate stimulates erythrophagocytosis in BM macrophages, which in turn provide iron to HSCs, thus favoring their differentiation at the expense of self-renewal.

In conclusion, present and future advances in our knowledge of the interaction between iron metabolism and erythropoiesis are expected to translate into better therapies for patients with disordered erythropoiesis.

## Data Availability

Not applicable.
